# Daily physical activity and prognostic implications in patients with heart failure: an accelerometer study

**DOI:** 10.1007/s00392-024-02508-0

**Published:** 2024-09-02

**Authors:** Andreas Bugge Tinggaard, Lotte Sørensen, Kristian Vissing, Niels Jessen, Helene Nørrelund, Henrik Wiggers

**Affiliations:** 1 Department of Cardiology, Aarhus University Hospital, Palle Juul-Jensens Boulevard 99, 8200 Aarhus N, Denmark; 2 Department of Clinical Medicine, Aarhus University, Palle Juul-Jensens Boulevard 99, 8200 Aarhus N, Denmark; 3 Department of Physiotherapy and Occupational Therapy, Aarhus University Hospital, Palle Juul-Jensens Boulevard 99, 8200 Aarhus N, Denmark; 4 Section for Sport Science, Department of Public Health, Aarhus University, Bartholins Allé 2, 8000 Aarhus C, Denmark; 5 Steno Diabetes Center Aarhus, Aarhus University Hospital, Palle Juul-Jensens Boulevard 11, 8200 Aarhus N, Denmark; 6 Department of Biomedicine, Aarhus University, Hoegh-Guldbergsgade 10, 8000 Aarhus C, Denmark; 7 Department of Clinical Pharmacology, Aarhus University Hospital, Palle Juul-Jensens Boulevard 99, 8200 Aarhus N, Denmark

**Keywords:** Accelerometry, Daily physical activity, Exercise testing, Quality of life, Patient-reported outcome

## Abstract

**Background:**

Physical activity (PA) measured by accelerometry is proposed as a novel trial endpoint for heart failure (HF). However, standardised methods and associations with established markers are lacking. This study aimed to examine PA measurements and accelerometer repeatability in patients with HF and age- and sex-matched controls, and study correlations with established prognostic HF markers, body composition, and quality of life (QoL).

**Methods:**

Accelerometry was performed in 105 patients with HF with left ventricular ejection fraction (LVEF) ≤ 40% and in 46 controls. Participants also underwent dual X-ray absorptiometry, cardiopulmonary exercise testing, a six-minute walking test (6MWT), echocardiography, and NT-proBNP measurement, and completed a QoL questionnaire.

**Results:**

Average acceleration was markedly reduced in patients with HF compared with healthy controls (16.1 ± 4.8 mg vs 27.2 ± 8.5 mg, *p* < 0.001). Healthy controls spent a median daily 56 min (IQR 41–96 min) in moderate-to-vigorous PA (MVPA), whereas HF patients spent only 12 min (IQR 6–24) in MVPA. In HF patients, average acceleration correlated moderately with 6MWT (*R* = 0.41, *p* < 0.001) and maximal oxygen uptake (peak *V*O_2_) (*R* = 0.36, *p* < 0.001) but not with NT-proBNP, LVEF, or QoL. Patients in NYHA class II showed a higher average acceleration than patients in NYHA III (16.6 ± 4.9 mg vs 14.0 ± 3.6 mg, *p* = 0.01).

**Conclusions:**

Daily PA was severely reduced in patients with HF compared with healthy controls. In HF patients, we found moderate correlations of accelerometer measurements with markers of physical capacity but not with LVEF or NT-proBNP.

**Trial registration:**

NCT05063955. Registered 01 June 2021—retrospectively registered.

**Graphical abstract:**

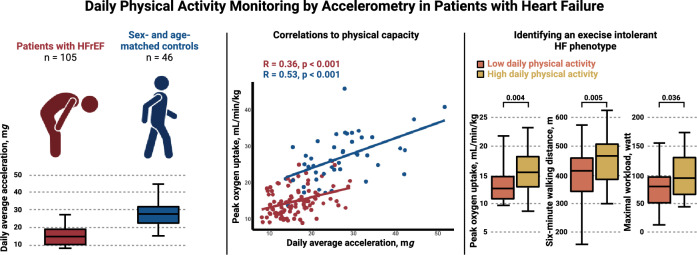

**Supplementary Information:**

The online version contains supplementary material available at 10.1007/s00392-024-02508-0.

## Background

Promoting physical activity (PA) in heart failure (HF) is an important treatment strategy as regular PA is associated with a lower risk of hospitalisation and death [1–3]. Furthermore, PA decreases disease progression, the risk of functional limitations, and loss of independence [1, 4]. Hence, monitoring, analysing, and reporting PA in patients with HF may enhance care for these patients, but only a few recommendations exist on this topic [5]. Accelerometry constitutes a more recent approach to obtaining objective PA measurements and is considered superior to self-reported PA [6]. The accelerometer provides accurate measurements of PA intensity with a high temporal resolution under free-living conditions [7]. Recently, daily PA assessed by accelerometry has been proposed as a novel endpoint in HF trials [8, 9]. For accelerometry to be a comprehensible method for daily PA assessment in HF research, studies linking accelerometer measurements to established clinical endpoints are needed. Traditionally, the physical condition of patients with HF has been evaluated using physical capacity tests such as a cardiopulmonary exercise test or a six-minute walking distance (6MWD) test—both of which are of prognostic value [3]. Moreover, low daily PA has been associated with a higher symptom burden and decreased quality of life (QoL) in patients with HF [10]. To improve the validity of PA measurements, usage of tri-axial accelerometers is recommended [5]. Additionally, accelerometers providing raw acceleration data and allowing for post-monitoring data processing are preferred over accelerometers based on proprietary algorithms that convert data into arbitrary PA metrics such as steps, “activity counts”, or calories [11]. This raw data approach enhances reproducibility and external validity.

Only a few studies have examined the relationships between PA, physical capacity, and QoL in patients with HF [12–14]. To our knowledge, no previous studies have used tri-axial, wrist-worn accelerometers providing raw acceleration data that may provide pragmatic and comprehensive insight into daily PA in patients with HF. Thus, the aims of this study were, first, to characterise PA measurements in patients with HF with reduced ejection fraction (HFrEF) compared with age- and sex-matched healthy controls; second, in patients with HFrEF, to study the relationships between objectively measured PA and well-established markers of HF severity, physical capacity, body composition, and QoL; third, to identify and characterise the group of patients with HFrEF and low PA. Finally, we aimed to evaluate the repeatability of PA measurements.

## Methods

### Study participants

Eligible patients with HF were assessed at a screening visit in the HF outpatient clinic at Aarhus University Hospital, Denmark, between April 2020 and August 2023. The inclusion criteria were: (1) an HF diagnosis according to European Society of Cardiology guidelines [1], (2) left ventricular ejection fraction (LVEF) ≤ 40%, (3) New York Heart Association (NYHA) functional class II–IV, and (4) clinically stable without changes in cardiac medications for 4 weeks prior to inclusion. The exclusion criteria were: (1) severe lung disease with a forced expiratory volume in the first second ≤ 40%, (2) severe musculoskeletal or neurological disability, and (3) cancer requiring active treatment.

Age- and sex-matched healthy controls were recruited via community newspaper advertisement. The exclusion criteria were similar to those of patients with HF and included the absence of any history of ischemic heart disease, valvular heart disease, cardiac arrhythmias, or HF. In addition, the controls were required to have a normal electrocardiogram and echocardiography, and a normal blood sample screening for anaemia and thyroid disease. The study was conducted at Aarhus University Hospital, Denmark, in compliance with the Declaration of Helsinki, and was approved by the Research Ethics Committee of the Central Denmark Region. All participants provided written informed consent before enrolment.

### Study design

All participants were scheduled for two study visits. The first visit included fasting blood samples, a dual X-ray absorptiometry (DXA), maximal muscle strength tests, completion of questionnaires, and a 6MWD test. At the first visit, all participants received an accelerometer and wore it for 12 days. After 2–6 weeks at the second visit, the participants underwent echocardiography (unless patients were stable and the most recent examination had been performed within 6 months) and a cardiopulmonary exercise test (CPET).

### Accelerometry

The Axivity AX3 (Axivity, Newcastle upon Tyne, UK) accelerometer was used to assess 24-h activity cycles. The AX3 triaxial monitor records acceleration in the vertical, anteroposterior, and mediolateral axes. The AX3 has been field validated with doubly labelled water (*r* = 0.87–0.91) [15] and laboratory validated (*r* = 0.63–0.87) [16]. Participants were instructed to wear the accelerometer on their nondominant wrist for 12 consecutive days and to remove it only during water activities.

Accelerometers were programmed to store accelerations at 100 Hz with a dynamic range of 8 g. Raw data were downloaded as “.cwa” files and processed with the R software using the “GGIR” 2.6-2 package [17]. In brief, data processing included autocalibration according to the local gravity [18], detection of sustained abnormally high values, detection of nonwear, and calculation of the average magnitude of dynamic acceleration corrected for gravity generating the metric Euclidean Norm Minus One expressed in milligravitational units (m*g*). Nonwear detection has previously been described [19]. The exact script used for raw data processing is provided in Supplementary (eSupplementary 1). Only participants providing at least 7 days of ≥ 16 h of daily wear time were included in the study.

The following outcomes were calculated and averaged across all valid days: average acceleration, intensity gradient, daily time spent in moderate-to-vigorous PA (MVPA), daily time spent in light PA (LPA), and daily time spent in sedentary activity (SED). The intensity gradient reflects the distribution of acceleration intensity across the 24-h cycle and has been described previously [20]. In brief, the gradient describes the negative curvilinear relationship between PA intensity and time accumulated at that intensity during a 24-h cycle. Together, the average acceleration and intensity gradient provide a description of a person’s entire activity profile and the relative weight of intensity and volume of activity. We used a pragmatic definition of time spent in SED as activity < 30 mg, time spent in LPA as activity at 30–99 mg, and MVPA as activity ≥ 100 mg. These cut-offs are based on laboratory studies [21, 22], and are widely used in accelerometry studies [23–25].

To assess the day-to-day variability and variation between the first 5 days and the last 5 days of accelerometry, single-day summaries were extracted from the accelerometry. First, we compared the average acceleration on odd and even measurement days. Second, we compared the average acceleration from the first five complete cycles of 24-h accelerometry and the last five complete cycles of 24-h accelerometry. To assess within the HF population whether a low PA group would demonstrate decreased physical capacity, decreased QoL, and lower muscle mass than a high PA group, the HF population was divided into a low PA group and a high PA group based on the median average acceleration with a cut-off at 15.1 mg.

### Functional tests

CPET was performed using a cycle ergometer (JAEGER Vyntus CPX, Vyaire Medical, IL, USA) and a standardised ramp protocol with an incrementally increased workload from 0 W to exhaustion for a projected 8- to 12-min work period. Gas exchange was measured continuously during exercise to obtain maximal oxygen consumption (peak *V*O_2_), maximal carbon dioxide production (peak *V*CO_2_), and respiratory exchange ratio. The 6MWD was assessed on an indoor 30-m course.

### Muscle strength

Maximal unilateral isometric and isokinetic knee-extension strength was tested in a dynamometer (CON-TREX, Physiomed, Schnaittach, Germany). The highest peak torque of three voluntary contractions of the dominant leg was defined as maximal isometric strength. An isometric handgrip strength test was performed using a handheld dynamometer (Charder Medical, Taichung City, Taiwan). Maximal handgrip strength was defined as the best result of three attempts with the participant’s dominant hand.

### Body composition

Body composition was assessed using DXA (Hologic, MA, USA). The following measurements were obtained: total body fat mass, fat percentage, and appendicular lean mass. Appendicular lean mass was indexed to body mass index (BMI) (ALMi). Men and women have different cutoff values for low muscle mass based on the ALMi. In this study, we used previously suggested cutoffs for detecting low muscle mass; ALMi < 0.789 for men and ALMi < 0.512 for women [26, 27].

### Questionnaires

Self-reported QoL was measured in patients with HF using the standardised Minnesota Living with Heart Failure Questionnaire (MLHFQ).

### Statistical analysis

All statistical analyses were performed using R (version 4.0.3, R Foundation for Statistical Computing, Vienna, Austria. URL http://r-project.org) and RStudio (version 2022.07.1, RStudio: Integrated Development for R, Boston, MA, USA. URL http://www.rstudio.com/). Data were assessed for normality by inspecting QQ plots and histograms.

Day-to-day agreement and variation between the first 5 days and the last 5 days of accelerometry were assessed by Bland–Altman plots and the intraclass correlation coefficient. Participant characteristics were presented as mean ± standard deviation or median with interquartile range (IQR for continuous variables and frequency (percentages) for categorical variables. Between-group differences (HF vs healthy controls, HF low PA vs HF high PA) were tested with independent sample *t* test or Mann–Whitney *U* test for continuous variables and *χ*^2^ test for categorical variables. To compare daily physical activity between patients with HF and healthy controls, we used analysis of covariance to adjust for potential confounding variables such as age, sex, and BMI. Bivariate correlations of physical capacity tests, HF parameters, and accelerometer measurements were assessed using Pearson or Spearman rank correlation. For variables not normally distributed, logarithmic transformation was performed prior to the correlation analysis. Additionally, univariable and multivariable regression models were employed to further support correlations between physical capacity, HF parameters, and accelerometer measurements, with average acceleration and time spent in MVPA as the dependent variables. To avoid collinearity, specific variables were removed from the multivariable regression models. Statistical significance was considered as a *p* value < 0.05.

## Results

**Fig. 1:**
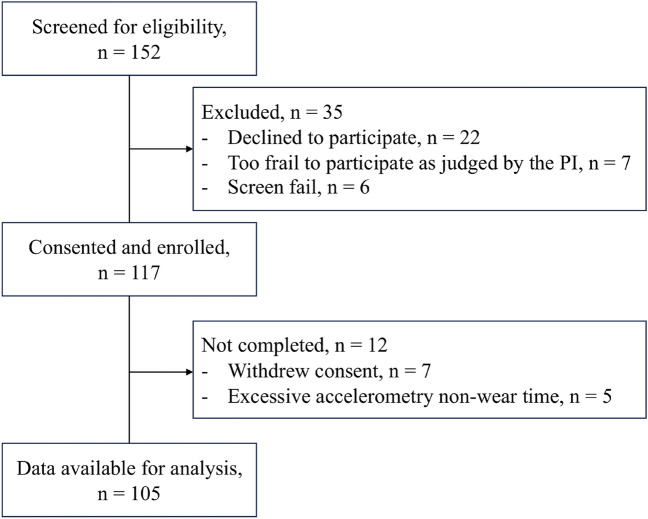
Flow diagram of the enrolment of patients with heart failure. In total, 152 patients with heart failure with reduced ejection fraction were screened in the heart failure clinic, 117 were enrolled and 105 patients with heart failure completed the examinations providing data for analysis. *PI* principal investigator

We performed accelerometry in 156 participants of whom 105 patients with HF and 46 healthy controls had sufficient PA data. Figure 1 shows the enrolment of patients with HF. Five patients with HF were excluded due to excessive nonwear time (for baseline characteristics of excluded patients, see eTable 1 in the Supplementary). Patients with HF and controls did not differ with respect to age (72 ± 8 vs 71 ± 5 years) or sex (24% vs 26% females). Patients with HF were characterised by a higher BMI and prevalence of diabetes and chronic kidney disease (Table 1). In the HF group, mean LVEF was 31 ± 7%, and median NT-proBNP was 886 ng/L (IQR 376–1672 ng/L). The median HF duration was 59 months (IQR 11–110 months).

**Table 1:** Study participant characteristics

Characteristic	Control *N* = 46	HF, highPA *N* = 54	HF, lowPA *N* = 51	Control vs heart failure	HF, lowPA vs HF, highPA
Age	71 ± 5	72 ± 8	72 ± 7	0.5	0.8
Sex, female	12 (26%)	15 (28%)	10 (20%)	0.8	0.3
NYHA class				–	0.2
NYHA II	–	46 (85%)	38 (75%)		
NYHA III	–	8 (15%)	13 (25%)		
Duration of HF, months	–	46 (7–103)	74 (20–129)	–	0.14
Etiology of HF, ischemic	–	28 (54%)	30 (60%)	–	0.5
BMI	25 ± 4	28 ± 5	30 ± 6	**< 0.001**	0.2
MLHFQ score	–	28 ± 17	30 ± 20	–	0.7
Comorbidities					
Hypertension	15 (33%)	21 (40%)	25 (49%)	0.2	0.3
Dyslipidemia	28 (62%)	36 (67%)	28 (56%)	0.9	0.3
Diabetes	2 (4%)	8 (15%)	16 (32%)	**0.005**	**0.029**
CKD	1 (2.2%)	13 (24%)	14 (27%)	** < 0.001**	0.7
Treatment					
Beta-blocker	0 (0%)	50 (93%)	50 (98%)	–	0.4
ACEi/ARB/ARNi	9 (20%)	40 (74%)	45 (88%)	** < 0.001**	0.083
MRA	0 (0%)	31 (57%)	30 (59%)	–	0.9
SGLT2i	0 (0%)	24 (44%)	21 (41%)	–	0.7
Diuretics	0 (0%)	42 (78%)	34 (67%)	–	0.2
CRT	–	19 (37%)	18 (37%)	–	0.9
ICD	–	30 (56%)	29 (59%)	–	0.7
Laboratory analyses					
Creatinine µmol/L	76 (68–83)	91 (80–106)	105 (80–129)	** < 0.001**	0.2
NT-ProBNP, ng/L	91 (58–201)	832 (409–1,487)	924 (325–1,767)	** < 0.001**	0.9
Haemoglobin, mmol/L	9.0 (8.7–9.5)	8.8 (8.1–9.2)	8.7 (8.2–9.2)	**0.006**	0.4
Ferritin, µg/L	103 (69–185)	105 (53–191)	144 (42–189)	0.9	0.4
Echocardiography					
Left ventricular ejection fraction, %	57 ± 5	30 ± 7	33 ± 7	** < 0.001**	0.12
Global longitudinal strain, –%	18.8 ± 2.2	9.1 ± 2.6	9.8 ± 2.7	** < 0.001**	0.3
Physical capacity					
Peak *V*O_2_, mL/min/kg	27 ± 7	16 ± 4	14 ± 4	** < 0.001**	**0.004**
Absolute *V*O_2_, mL/min	1992 ± 437	1337 ± 437	1207 ± 332	** < 0.001**	0.15
Maximal workload, W	177 ± 39	99 ± 38	83 ± 37	** < 0.001**	0.036
6-min walking distance, m	615 ± 78	458 ± 86	390 ± 110	** < 0.001**	**0.005**
Maximal isometric knee-extensor strength, Nm	132 ± 37	111 ± 42	100 ± 37	** < 0.001**	0.2
Handgrip strength, kg	37 ± 9	32 ± 10	32 ± 9	**0.003**	0.7
Skeletal muscle					
Muscle wasting group				** < 0.001**	**0.014**
Low muscle mass	7 (15%)	19 (36%)	30 (60%)		
Preserved muscle mass	39 (85%)	34 (64%)	20 (40%)		

Bold *p*-values are < 0.05

Values are mean ± standard deviation, median (interquartile range) or *n* (%) as appropriate. Between-group comparisons are denoted with *p* values for healthy controls vs patients with heart failure and patients with heart failure in the low vs high physical activity group. Values are mean ± standard deviation, median (interquartile range) or n (%) as appropriate

*ACEi* angiotensin-converting enzymes inhibitors, *BMI* Body Mass Index, *CKD* chronic kidney disease, *CRT* cardiac resynchronization therapy, *HF* heart failure, *ICD* implantable cardioverter-defibrillator, *MLHFQ* Minnesota Living with Heart Failure Questionnaire, *MRA* mineralocorticoid receptor antagonists, *MVPA* moderate-to-vigorous physical activity, *NT-proBNP* N-terminal pro-B-type natriuretic peptide, *NYHA* New York Heart Association, *SGLT2i* sodium-glucose cotransporter-2 inhibitors, *Peak VO*_*2*_ peak oxygen consumption

### Measures of daily physical activity in patients with heart failure and matched controls

Average acceleration was lower in patients with HF than in healthy controls (16.1 ± 4.8 mg vs 27.2 ± 8.5 mg, *p* < 0.001), and in contrast to that in healthy controls, a lower intensity gradient among patients with HF suggested less time accumulated at midrange and higher intensities (− 3.9 ± 0.6 vs − 3.1 ± 0.5, *p* < 0.001). The results can be found in Table 2. This was supported by analysis of time spent at various intensity levels. Median daily time spent in MVPA for patients with HF was 12 min (IQR 6–24 min), whereas median time spent in MPVA for healthy controls was 56 min (IQR 41–96 min, *p* < 0.001). Daily time spent in LPA was 216 min (± 84 min) for patients with HF and 280 min (± 80 min) for healthy controls (*p* < 0.001). Additionally, patients with HF spent significantly more time in sedentary state than healthy controls did (801 ± 135 min vs 643 ± 116 min, *p* < 0.001), and less time sleeping (398 ± 101 min vs 440 ± 55 min, *p* = 0.011). The results were consistent after adjustment for sex, age, and BMI.

**Table 2:** Daily physical activity measurements

Measurement	Control, *n* = 46	Heart failure, *n* = 105	*p* value*	*p* value^†^	*p* value^‡^
Average acceleration, m*g*	27.2 ± 8.5	16.1 ± 4.8	** < 0.001**	** < 0.001**	** < 0.001**
Intensity gradient	− 3.1 ± 0.5	− 3.9 ± 0.6	** < 0.001**	** < 0.001**	** < 0.001**
Time spent in SED, min	643 ± 116	801 ± 135	** < 0.001**	** < 0.001**	** < 0.001**
Time spent in LPA, min	280 ± 80	216 ± 84	** < 0.001**	** < 0.001**	** < 0.001**
Time spent in MVPA. min	56 (41–96)	12 (6–24)	** < 0.001**	** < 0.001**	** < 0.001**
Time spent sleeping, min	440 ± 55	398 ± 101	**0.011**	**0.013**	0.06

Bold *p*-values are < 0.05

Measurements are mean ± standard deviation or median (interquartile range)

*LPA* light physical activity, *mg* milligravitational units, *MVPA* moderate-to-vigorous physical activity, *SED* sedentary activity

*Unadjusted

^†^Adjusted for sex and age

^‡^Adjusted for sex, age, and BMI

### Correlations between daily physical activity and physical capacity, body composition, heart failure prognostics, and quality of life

**Fig. 2:**
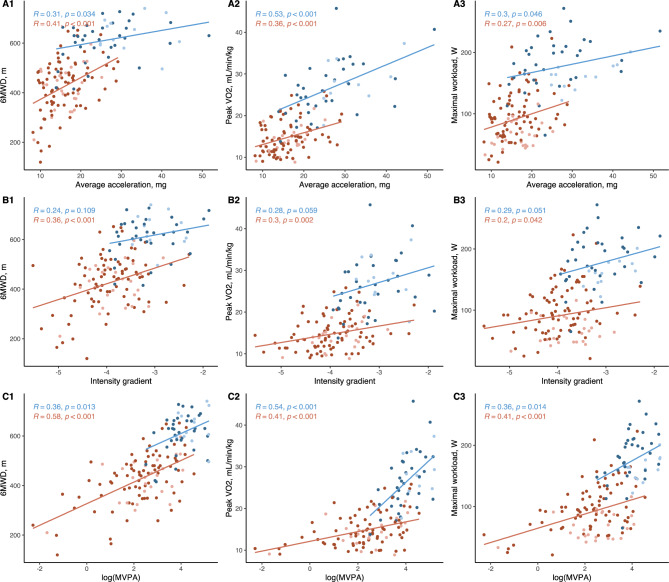
Correlation plots between accelerometer measurements and physical capacity measurements in patients with heart failure. A1, A2, and A3 show correlations between average acceleration and six-minute walking distance, peak oxygen consumption, and maximal workload of the cardiopulmonary exercise test. B1, B2, and B3 show correlations between the intensity gradient, six-minute walking distance, peak oxygen consumption, and maximal workload of the cardiopulmonary exercise test. C1, C2, and C3 show correlations between the logarithm of daily time spent in moderate-to-vigorous physical activity, six-minute walking distance, peak oxygen consumption, and maximal workload of the cardiopulmonary exercise test. The Pearson correlation coefficients (*R*) and *p* values are shown. Lighter red dots represent women with heart failure, and darker red dots represent men with heart failure. Similarly, lighter blue dots indicate control women, whereas darker blue dots indicate control men. The solid lines represent the linear regression lines for the entire heart failure and control group, respectively. *6MWD* six-minute walking distance, *mg* milligravitational units, *MVPA* moderate-to-vigorous physical activity, *peak V*O_2_ peak oxygen consumption

**Fig. 3:**
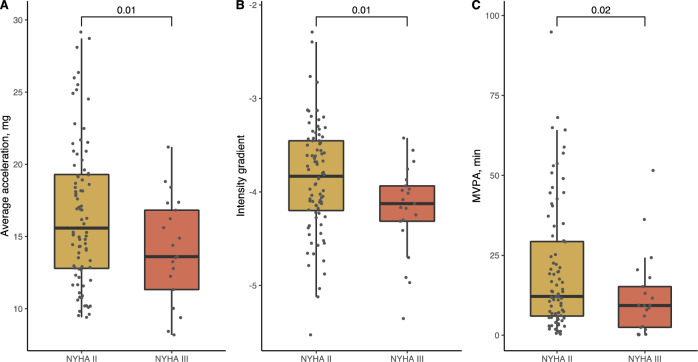
Boxplots of physical activity measurements in patients with heart failure, NYHA II vs NYHA III. The figure shows boxplots of **A** average acceleration, **B** intensity gradient and **C** daily time spent in MVPA. The p values for the comparison between the two groups are shown. *mg* milligravitational units, *MVPA* moderate-to-vigorous physical activity, *NYHA* New York Heart Association

In patients with HF, average acceleration was only moderately correlated with 6MWD (*R* = 0.41, *p* < 0.001), peak *V*O_2_ (*R* = 0.36, *p* < 0.001), and maximal workload at CPET (*R* = 0.27, *p* = 0.006). Similarly, we found that the intensity gradient was moderately correlated with 6MWD (*R* = 0.36, *p* < 0.001) and peak *V*O_2_ (*R* = 0.3, *p* = 0.002), and weakly significantly correlated with maximal workload (*R* = 0.2, *p* = 0.042) (Fig. 2). No correlations were found between average acceleration and muscle strength, QoL, LVEF, or NT-ProBNP (eFigure 1 in the Supplementary). Compared with patients in NYHA class III, patients with HF in NYHA class II showed a higher average acceleration (16.6 ± 5.0 mg vs 14.0 ± 3.6 mg, *p* = 0.01), a greater intensity gradient (− 3.9 ± 0.6 vs − 4.2 ± 0.5, *p* = 0.01) and more daily minutes spent in MVPA (12, IQR 6–29 min vs 9, IQR 2–15 min, *p* = 0.02) (Fig. 3). The logarithm of time spent in MVPA was moderately correlated with both 6MWD (*R* = 0.58, *p* < 0.001), peak *V*O_2_ (*R* = 0.41, *p* < 0.001), and maximal workload (*R* = 0.41, *p* < 0.001) (Fig. 2). We found no correlations between other PA measurements (LPA and SED) and exercise capacity.

The univariable and multivariable regression models supported these associations in patients with HF (Table 3). In the univariable regression, 6MWD, peak *V*O_2_, and maximal workload predicted average acceleration and time spent in MVPA (all *p* values < 0.01), whereas no associations were found between HF parameters, average acceleration, and MVPA. In the multivariable regression, after adjusting for age, sex, and HF parameters, the predictive value of 6MWD and peak *V*O_2_ remained highly statistically significant (all *p* values < 0.01).

**Table 3:** Univariable and multivariable regression models with average acceleration and time spent in MVPA as outcomes in the cohort of patients with heart failure (*n* = 105)

	Univariable			Multivariable, 6MWD			Multivariable, peak *V*O_2_		
	Beta	95% CI	*p* value	Beta	95% CI	*p* value	Beta	95% CI	*p* value
Average acceleration									
Age	− 0.05	− 0.08, 0.19	0.4	0.06	− 0.08, 0.19	0.4	0.00	− 0.13, 0.14	> 0.9
Sex, male	− 0.27	− 2.5, 1.7	0.7	− 0.40	− 2.5, 1.7	0.7	− 0.37	− 2.6, 1.8	0.7
6MWD*	0.02	0.01, 0.03	** < 0.001**	0.02	0.01, 0.03	** < 0.001**			
Peak * V*O_2_*	0.45	0.22, 0.69	** < 0.001**				0.42	0.15, 0.70	**0.003**
Maximal workload*	0.03	0.01, 0.06	**0.006**						
Duration of HF^†^	− 0.10	− 0.20, 0.13	0.7	− 0.03	− 0.20, 0.13	0.7	− 0.02	− 0.20, 0.16	0.8
LVEF*	− 0.04	− 0.14, 0.19	0.8	0.02	− 0.14, 0.19	0.8	− 0.01	− 0.18, 0.16	> 0.9
GLS*	0.00								
NT-proBNP^‡^	− 0.03	− 0.03, 0.06	0.5	0.01	− 0.03, 0.06	0.5	0.00	− 0.05, 0.05	> 0.9
Time spent in MVPA									
Age	− 0.55	− 1.0, − 0.07	**0.026**	− 0.14	− 0.67, 0.38	0.6	− 0.34	− 0.84, 0.16	0.2
Sex, male	− 1.9	− 10, 6.7	0.7	− 2.3	− 11, 5.9	0.6	− 2.3	− 11, 6.0	0.6
6MWD*	0.08	0.05, 0.11	** < 0.001**	0.08	0.04, 0.12	** < 0.001**			
Peak *V*O_2_*	2.1	1.2, 3.0	** < 0.001**				2.0	1.0, 3.0	** < 0.001**
Maximal workload*	0.16	0.07, 0.25	** < 0.001**						
Duration of HF^†^	− 0.19	− 0.83, 0.46	0.6	0.09	− 0.55, 0.74	0.8	0.23	− 0.44, 0.89	0.5
LVEF*	− 0.22	− 0.76, 0.32	0.4	0.12	− 0.52, 0.76	0.7	0.06	− 0.58, 0.69	0.9
GLS*	0.06	− 1.5, 1.6	> 0.9						
NT-proBNP^‡^	− 0.13	− 0.28, 0.03	0.11	0.04	− 0.15, 0.22	0.7	0.00	− 0.18, 0.17	> 0.9

Bold *p*-values are < 0.05

*6MWD* 6-min walking distance, *GLS* global longitudinal strain, *HF* heart failure, *LVEF* left ventricular ejection fraction, *NT-proBNP* N-terminal pro-B-type natriuretic peptide, *peak VO*_*2*_, peak oxygen consumption

*1-unit incremental

^†^1-year incremental

^‡^100-unit incremental

In healthy controls, we found similar significant correlations between physical capacity tests and accelerometer measurements except for the intensity gradient (Fig. 2).

### Low physical activity versus high physical activity in patients with heart failure

**Fig. 4:**
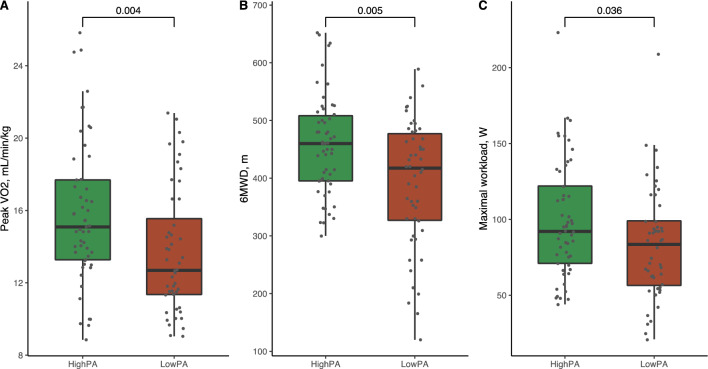
Boxplots of physical capacity measurements in a high physical activity group vs a low physical activity group. The figure shows boxplots of **A** peak oxygen consumption, **B** six-minute walking distance, and **C** maximal workload of the cardiopulmonary exercise test. The *p* values for the comparison between the two groups are shown. *6MWD* six-minute walking distance, *PA* physical activity, *peak V*O_2_ peak oxygen consumption

Based on the median average acceleration of 15.1 mg, patients with HF were divided into a low PA group and a high PA group. Table 1 summarises patient characteristics and differences in echocardiographic measurements, HF markers, QoL, and physical capacity. No differences were observed in age or sex between the groups. Patients in the low PA group were characterised by a lower peak *V*O_2_ (14 ± 4 mL/min/kg vs 16 ± 4 mL/min/kg, *p* = 0.004) and a shorter 6MWD (458 ± 86 m vs 390 ± 110 m, *p* = 0.005). Patients in the high PA group achieved a higher maximal workload at CPET than patients in the low PA group (99 ± 38 W vs 83 ± 37 W, *p* = 0.036) (Fig. 4).

Although more patients in the low PA group presented with low muscle mass assessed by the ALMi than patients in the high PA group did (60% vs 36%, *p* = 0.014), we found no differences in handgrip (32 ± 10 kg vs 32 ± 9 kg, *p* = 0.7) or knee-extensor strength (111 ± 42 Nm vs 100 ± 37 Nm, *p* = 0.2). No differences were found in NYHA class (25% in NYHA class III in the low PA group vs 15% in the high PA group, *p* = 0.2) or MLHFQ score (30 ± 20 vs 28 ± 17, *p* = 0.7). Patients with low PA did not differ with respect to LVEF (30 ± 7% vs 33 ± 7%, *p* = 0.12) or NT-ProBNP (832, IQR 409–1487 ng/L vs 925, IQR 325–1767 ng/L, *p* = 0.9) (Table 1).

### Variability and repeatability of accelerometry measurements

**Fig. 5:**
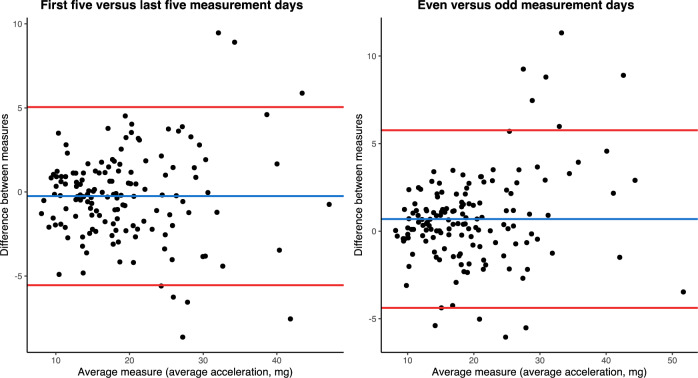
Bland–Altman plots demonstrating the agreement of measurements. Agreement for the first 5 days versus the last 5 days of accelerometry and the agreement for even and odd measurement days are shown to assess the day-to-day variability. The difference between measurements is shown on the *y*-axis and is plotted against the average acceleration on the *x*-axis. The mean difference is indicated by the blue line and the 95% confidence interval is indicated by the red lines. *mg* milligravitational units

We found no difference in average acceleration between weekdays and weekend days in patients with HF (16.1 mg vs 15.1 mg, *p* = 0.13) or in healthy controls (26.3 mg vs 27.5 mg, *p* = 0.57). Figure 5 shows Bland–Altman plots of the agreement between odd and even measurement days to assess the day-to-day variability and variation between the first five complete cycles of 24-h accelerometry and the last five complete cycles of 24-h accelerometry. The intraclass correlation coefficients for odd days vs even days were 0.95 (95% confidence interval (CI) 0.92–0.96, *p* < 0.001) and 0.94 (95% CI 0.92–0.96, *p* < 0.001) for the first 5 days vs the last 5 days of accelerometry.

## Discussion

In this study, we compared PA measurements in patients with HFrEF with those of age- and sex-matched healthy controls. Additionally, in patients with HF, we investigated the relationships between PA measurements and established markers of physical capacity and prognosis. This study produced several main findings: first, the repeatability of PA measurements was high. Second, the daily PA in patients with HFrEF was severely reduced compared with that of healthy controls, and the time spent in MVPA represented a key difference between the PA of patients with HF and that of healthy controls. Third, in patients with HF, we found only moderate correlations between accelerometer measurements, peak *V*O_2_, and 6MWD; and no significant correlations with other traditional HF prognostic markers, such as LVEF and NT-ProBNP. Finally, a low PA phenotype among patients with HF was characterised by poor physical capacity.

### Physical activity measurements in patients with heart failure and healthy controls

To our knowledge, this is the first study to compare daily PA between patients with HFrEF and sex- and age-matched healthy controls using accelerometry. A previous accelerometry study conducted using data from the UK Biobank showed an average acceleration of 23.7 mg in patients with HF and 28.1 mg in healthy controls [28]. The HF population was younger (66 years) and was not restricted to patients with HFrEF, which probably explains the higher average acceleration than recorded in the present study. Another UK Biobank study reported a similar average acceleration in 53 patients with HF of 21.5 mg as compared with 27.9 mg in participants without chronic diseases [23]. However, both studies were retrospective, registry-based, and lacked clinical participant data. In the present study, we support PA data with information on physical capacity, HF markers such as LVEF, NT-ProBNP, and also NYHA class, body composition, and QoL. We demonstrated a severely decreased PA level in patients with HF with a median of 12 min spent in MVPA for patients with HF compared with 56 min for healthy controls. In comparison, the World Health Organization recommends a minimum of 30 min of MVPA per day for healthy adults over 65 years of age and adults living with chronic conditions [29]. This was achieved only by 21% of the patients with HF as opposed to 89% of the healthy controls. Moreover, patients with HF spent more than 13 h daily in a sedentary state, but only 6.6 h sleeping. In contrast, healthy controls spent 10.7 h in a sedentary state and 7.3 h sleeping. Thus, our findings document that particularly time spent in MVPA and a sedentary state represent key differences between the PA of patients with HF and that of healthy controls.

Patients with HF demonstrated a higher prevalence of comorbidities and had higher BMI. Multimorbidity contributes to low PA [30], and, aside from the HF condition, the multimorbid characteristics of patients with HF may additionally reduce daily PA. As expected, healthy controls performed better on the CPET and the 6MWD test, and they exhibited higher muscle strength than patients with HF. Hence, this study demonstrated severely reduced PA and physical capacity in patients with HF.

### Physical activity and known risk markers in heart failure

This study was the first to investigate correlations between objectively measured PA and well-established markers of exercise capacity in patients with HFrEF. Overall, we observed only moderate correlations. These findings have various clinical implications. CPET is the gold standard for measuring maximal exercise capacity and integrates the maximal functional capacity of the cardiac, respiratory, vascular, and musculoskeletal systems. This functional chain has numerous regulatory links from impaired cardiac function during exercise [31] and decreased skeletal muscle capillary density [32] to low peripheral oxygen extraction [33], all of which are compromised in patients with HF. The 6MWD reflects a submaximal exercise capacity under free-living conditions and may be more relevant for assessing patients’ daily life exercise capacity since they typically do not draw close to maximal effort. Both peak *V*O_2_ and 6MWD may be improved by exercise training and are known to provide strong prognostic information. We found only moderate correlations between accelerometer measurements, peak *V*O_2_, and 6MWD; and no correlations with other traditional HF prognostic markers such as LVEF or NT-ProBNP. These observations highlight that PA measurements may complement existing measures used by clinicians to assess the prognosis of patients with HF. Conversely, the only modest correlations observed between PA measurements with established HF risk markers underscore a need for more data to underpin the prognostic information of PA in patients with HFrEF.

### Characterisation of patients with heart failure with low daily physical activity

As explained previously, we used average acceleration to identify a low PA group among patients with HF. Although no differences were found in age, sex, NYHA class, NT-ProBNP, LVEF, or HF duration, patients in the low PA group were characterised by a significantly lower exercise capacity than patients in the high PA group. These findings support existing knowledge of the negative association between low daily PA and exercise intolerance in HF [34]. Low PA is a well-known risk factor for the development of HF [35], and in prospective studies, it is an obvious challenge to assess daily PA in patients before HF onset. Thus, patients with HF with low daily PA may represent a low PA phenotype throughout the continuum of HF stages [36]. This low PA phenotype possibly represents a high-risk group that is poorly identified by traditional prognostic markers.

Low skeletal muscle mass was more prevalent in the low PA group: 60% of the patients were categorised as having low muscle mass compared with only 36% in the high PA group. Although skeletal muscle wasting has previously been proposed as a key marker of exercise intolerance in HF [37], we observed no differences in handgrip or knee-extensor muscle strength between the low PA group and the high PA group. A link may exist between low PA, low skeletal muscle mass, and exercise intolerance in patients with HF, but studies examining skeletal muscle wasting in patients with HF are needed to assess this issue.

### Repeatability and variability of accelerometry in patients with heart failure

Accelerometry has been proposed as a novel endpoint in HF trials, but studies on accelerometry lack consistency in data collection and reporting methods. Furthermore, processing methods need to be improved as do calculating metrics based on raw acceleration [11, 38]. The present study proposes an approach to these issues by presenting average acceleration and the intensity gradient supported by time spent at various PA intensities. Repeatability in accelerometry has not previously been evaluated in patients with HF. In our study, a 12-day monitoring period showed no difference in average acceleration between weekdays and weekend days, and we reported a low day-to-day variability. Moreover, we found a low variation between the first 5 days and the last 5 days of accelerometry. The AX3 device provided raw acceleration data, which were processed in the open-source software R and RStudio using the GGIR package. This approach facilitates a high degree of transparency, reproducibility and external validation, allowing for the comparison of studies with diverse designs and settings, which was recently requested [38]. Thus, the applied accelerometer monitoring methodology proved to be robust and may serve as a framework for future accelerometry studies.

### Study limitations

Only 23% of the study participants were female; a well-recognised challenge in HF trials [39]. In national registries, 30% of the incident HFrEF population is female [40]. The mean age of participants in HF clinical trials is typically approximately 65 years [41], which contrasts with the mean age (70 years) at HF onset according to data from national registries [40]. Hence, our study population, with a mean age of 72 years, matches the real-life HF population. Finally, this study was performed in stable patients with symptomatic HF in NYHA classes II and III. During recruitment, several of the patients who declined or were too frail to participate were classified as NYHA class IV. Thus, whether the results apply to asymptomatic patients or patients with more advanced HF therefore remains unknown.

Though different device positions have been used in previous daily PA studies, the wrist-worn position is the most widely applied position due to high compliance and good accuracy [19, 22]. Furthermore, the autocalibration in the GGIR package has been developed and validated with raw data from wrist-worn accelerometers [18]. Thus, we used the wrist accelerometer position to assess the study participants’ daily PA, acknowledging that other methodologies exist.

Human activity expenditure is known to be complex, and wrist-worn tri-axial accelerometry may not distinguish between types of PA (e.g. bike riding, roller-skating, etc.). Although structured exercise bouts contribute to the total amount of daily PA, most PA volume in the general population consists of walking. Our approach to daily PA reporting allows for distinction between sedentary, light, moderate, and vigorous PA. Moreover, we propose the use of the intensity gradient to describe the relative weight of intensity and volume of activity.

We used established accelerometry cutoffs for sedentary, light, and moderate PA to compare patients with HF and healthy controls. Cutoffs are debated and need to be selected carefully in different cohorts, especially in older populations. To comply with the aim of comparing patients with HF with healthy controls, we chose the most widely used cutoffs for adults.

The moderate correlations observed do not imply a direct causal relationship between accelerometer measurements and physical capacity. Multiple unmeasured factors, both physiological, psychological, and socioeconomic, may contribute to these associations. Socioeconomic factors and environmental variables, such as the walkability of areas, work locations, and access to recreational facilities, can confound or be incorrectly associated with PA levels. Seasonal variations in outdoor activities and access to exercise locations were not considered in our study, which may influence PA patterns. Psychological factors, such as motivation and perceived exertion during exercise tests, along with the inherent intra-individual variability of these tests, can significantly influence performance outcomes and contribute to the weak correlations observed. The short monitoring period for accelerometry also introduces variability. The cross-sectional design employed in our study is a limitation for obtaining complete information regarding the participants’ lifelong exercise habits and broader lifestyle factors. Such information could be relevant for understanding the influence of prior exercise routines and lifestyle choices on the daily PA patterns observed. Future research should explore these factors and apply long-term daily PA monitoring to provide a more comprehensive understanding of daily PA in diverse settings.

## Conclusions

In patients with HF, we demonstrated that accelerometry is a robust method for assessing daily PA with high repeatability and low day-to-day variability. Daily PA was markedly lower in patients with HF than in healthy age- and sex-matched controls. We found moderate correlations between accelerometer measurements and well-established markers of exercise capacity but no correlations with LVEF, NT-proBNP, or QoL. Patients with NYHA class III symptoms had lower daily PA levels than patients in NYHA class II. Finally, patients with HF with low daily PA were characterised by exercise intolerance and low skeletal muscle mass. Overall, accelerometry in HF yields complementary information that cannot be ascertained by standard prognostic HF markers and tests.

## Supplementary Information

Below is the link to the electronic supplementary material.

Supplementary file1 (DOCX 288 kb)

## Data Availability

The data used and analysed during this study are available from the corresponding author on reasonable request.
