# Supplementary material for: Daily physical activity and prognostic implications in patients with heart failure: an accelerometer study
Source: Clin Res Cardiol. 2024 Sep 2;114(5):616–28. doi: 10.1007/s00392-024-02508-0 (PMC12058814; doi:10.1007/s00392-024-02508-0)
Supplement: Supplementary file 1 — Supplementary file1 (DOCX 288 kb) [file 392_2024_2508_MOESM1_ESM.docx]

**Supplementary**

**eSupplementary 1**

Applied script for raw data processing using the R-package “GGIR”. Data directories are anonymized.

library(GGIR)

g.shell.GGIR(

mode=c(1:5),

overwrite = TRUE,

datadir="~/***",

outputdir="~/***",

do.report=c(2, 4, 5),

#=====================

# Part 2

#=====================

strategy = 1,

acc.metric = "ENMO",

do.enmo = TRUE,

hrs.del.start = 0, hrs.del.end = 0,

maxdur = 0, includedaycrit = 16,

qwindow=c(0,24),

chunksize = 0.5,

qlevels = c(0, 0.1, 0.2, 0.3, 0.4, 0.5, 0.6, 0.7, 0.8, 0.9, 1),

ilevels=seq(0,400,by=50),

mvpathreshold =c(100),

iglevels = TRUE,

bout.metric = 4,

excludefirstlast = FALSE,

includenightcrit = 16,

#=====================

# Part 3 + 4

#=====================

def.noc.sleep = 1,

outliers.only = FALSE,

criterror = 4,

do.visual = TRUE,

#=====================

# Part 5

#=====================

threshold.lig = c(30), threshold.mod = c(100), threshold.vig = c(400),

boutcriter = 0.8, boutcriter.in = 0.9, boutcriter.lig = 0.8,

boutcriter.mvpa = 0.8, boutdur.in = c(1,10,30), boutdur.lig = c(1,10),

boutdur.mvpa = c(1),

part5_agg2_60seconds = TRUE,

includedaycrit.part5 = 2/3,

#=====================

# Visual report

#=====================

timewindow = c("WW"),

visualreport=TRUE)

**eTable 1**

Baseline characteristics of patients excluded due to excessive nonwear time and included patients

| **Characteristic** | **Excluded**  N = 5 | **Included in study**  N = 105 | **p-value** |
| --- | --- | --- | --- |
| Age | 77 ± 6 | 72 ± 7 | 0.12 |
| Sex, female | 1 (20%) | 25 (24%) | >0.9 |
| NYHA Class |  |  | 0.3 |
| NYHA II | 3 (60%) | 84 (80%) |  |
| NYHA III | 2 (40%) | 21 (20%) |  |
| Duration of HF, months | 19 (16-99) | 59 (11-110) | 0.6 |
| Etiology of HF, ischemic | 2 (40%) | 58 (57%) | 0.7 |
| BMI | 29 ± 4 | 29 ± 6 | >0.9 |
| MLHFQ score | 38 ± 37 | 29 ± 18 | 0.9 |
| **Comorbidities** |  |  |  |
| Hypertension | 3 (60%) | 46 (44%) | 0.7 |
| Dyslipidemia | 4 (80%) | 64 (62%) | 0.6 |
| Diabetes | 3 (60%) | 24 (23%) | 0.057 |
| CKD | 1 (20%) | 27 (26%) | >0.9 |
| **Treatment** |  |  |  |
| Beta-blocker | 5 (100%) | 100 (95%) | >0.9 |
| ACEi/ARB/ARNi | 4 (80%) | 85 (81%) | >0.9 |
| MRA | 1 (20%) | 61 (58%) | 0.2 |
| SGLT2i | 2 (40%) | 45 (43%) | >0.9 |
| Diuretics | 4 (80%) | 76 (72%) | >0.9 |
| CRT | 0 (0%) | 37 (37%) | 0.3 |
| ICD | 1 (25%) | 59 (57%) | 0.3 |
| **Laboratory analyses** |  |  |  |
| Creatinine micromol/L | 98 (97-102) | 94 (80-116) | 0.6 |
| NT-ProBNP, ng/L | 594 (479-1,133) | 886 (376-1,672) | 0.6 |
| Hemoglobine, mmol/L | 8.0 (7.7-8.3) | 8.7 (8.1-9.2) | 0.14 |
| Ferritin, microg/L | 100 (43-698) | 116 (49-189) | 0.9 |
| **Echocardiography** |  |  |  |
| Left ventricular ejection fraction, % | 34 ± 5 | 31 ± 7 | 0.4 |
| Global longitudinal strain, -% | 9.8 ± 2.4 | 9.5 ± 2.6 | 0.7 |
| **Physical capacity** |  |  |  |
| Peak VO2, mL/min/kg | 14 ± 3 | 15 ± 4 | >0.9 |
| Absolute VO2, mL/min | 1,225 ± 362 | 1,276 ± 394 | 0.8 |
| Maximal workload, W | 69 ± 20 | 92 ± 38 | 0.2 |
| 6-minute walking distance, m | 349 ± 82 | 426 ± 103 | 0.071 |
| Maximal isometric knee-extensor strength, Nm | 100 ± 56 | 106 ± 40 | >0.9 |
| Handgrip strength, kg | 31 ± 6 | 32 ± 10 | 0.9 |
| **Skeletal muscle** |  |  |  |
| Muscle wasting group |  |  | 0.7 |
| Low muscle mass | 3 (60%) | 48 (47%) |  |
| Preserved muscle mass | 2 (40%) | 55 (53%) |  |

Values are mean ± standard deviation, median (interquartile range) or n (%) as appropriate. Between-group comparisons are denoted with p-values for healthy controls vs patients with heart failure and patients with heart failure in the low vs high physical activity group.

Values are mean ± standard deviation, median (interquartile range) or n (%) as appropriate.

Abbreviations: ACEi, angiotensin-converting enzymes inhibitors; BMI, Body Mass Index; CKD, chronic kidney disease; CRT, cardiac resynchronization therapy; HF, heart failure; ICD, implantable cardioverter-defibrillator; MLHFQ, Minnesota Living with Heart Failure Questionnaire; MRA, mineralocorticoid receptor antagonists; MVPA, moderate-to-vigorous physical activity; NT-proBNP, N-terminal pro-B-type natriuretic peptide; NYHA, New York Heart Association; SGLT2i, sodium-glucose cotransporter-2 inhibitors; Peak VO_2_, peak oxygen consumption.

**eFigure 1:** Correlation plots between accelerometer measurements and muscle strength, NT-proBNP, LVEF and Minnesota Living with Heart Failure Questionnaire score in patients with heart failure

A1, A2, A3, and A4 show correlations between average acceleration and maximal knee-extensor strength, NT-proBNP, left ventricular ejection fraction, and Minnesota Living with Heart Failure Questionnaire score. B1, B2, B3, and B4 show correlations between the intensity gradient and the same parameters, and C1, C2, C3 and C4 show correlations for the logarithm of daily time spent in moderate-to-vigorous physical activity. Lighter red dots represent women with heart failure, and darker red dots represent men with heart failure. The Pearson correlation coefficients (R) and p-values are shown. The solid line represents the linear regression line for the correlation.
